# Black phosphorus for bone regeneration: Mechanisms involved and influencing factors

**DOI:** 10.1016/j.mtbio.2024.101211

**Published:** 2024-08-24

**Authors:** Ting Sun, Chufeng Li, Jiayi Luan, Fujian Zhao, Yanli Zhang, Jia Liu, Longquan Shao

**Affiliations:** a Foshan Stomatology Hospital & School of Medicine, Foshan University, Foshan, 528000, China; b School of Dentistry, Jinan University, Guangzhou, 510630, China; c Stomatological Hospital, Southern Medical University, Guangzhou, 510280, China

**Keywords:** Black phosphorus, Bone regeneration, Mechanism, Influencing factors

## Abstract

BP has shown good potential for promoting bone regeneration. However, the understanding of the mechanisms of BP-enhanced bone regeneration is still limited. This review first summarizes the recent advances in applications of BP in bone regeneration. We further highlight the possibility that BP enhances bone regeneration by regulating the behavior of mesenchymal stem cells (MSCs), osteoblasts, vascular endothelial cells (VECs), and macrophages, mainly through the regulation of cytoskeletal remodeling, energy metabolism, oxidation resistance and surface adsorption properties, etc. In addition, moderating the physicochemical properties of BP (i.e., shape, size, and surface charge) can alter the effects of BP on bone regeneration. This review reveals the underlying mechanisms of BP-enhanced bone regeneration and provides strategies for further material design of BP-based materials for bone regeneration.

## Graphical abstract

Schematic diagram of mechanisms of BP-promoted bone regeneration and the influencing factors regeneration.

**Figure:**
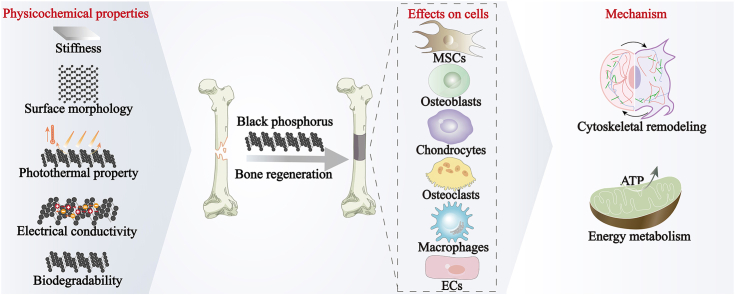

## 1 Introduction

Large bone defects caused by infection, trauma, tumors, bone joint disease, bone nonunion or delayed union have always been difficult to treat clinically [1]. Autologous or allogeneic bone grafts are able to repair bone defects more successfully. However, the need for additional surgery, the need for recovery of the donor area, and immune rejection have limited their application. Artificial bone substitution materials are now the mainstream way to repair bone defects, but the repair efficiency of using artificial bone substitution materials alone is low. However, the combined use of photothermal stimulation [2], electrical stimulation [3], immunomodulation [4]and other therapeutic methods can significantly enhance the repair effect of artificial bone substitute materials.

BP is an ideal material for bone regeneration mainly for its excellent photothermal property, electrical conductivity and immunoregulation capacity. BP is a two-dimensional (2D) crystal material with a unique layered structure and exhibits a bandgap that varies with the number of layers [5], making it more absorbent in the ultraviolet and NIR regions [6] compared to other two-dimensional materials, such as graphene and MXenes. After BP-based nanomaterials are implanted into the body, despite being covered by thick biological tissues, they still possess efficient photothermal conversion capabilities. The regular and stable local thermal environment created by them can activate various bone-forming proteins [7]. Recent studies also showed that BP incorporation obviously increase the electrical conductivity of hydrogels that promote bone regeneration [8]. It has also been reported BP can promote bone regeneration via modulating macrophage polarization [9,10].

Except for the above characteristics, BP also exhibit high degradability and biocompatibility [11,12], which are necessary for bone regeneration materials. The most important advantage is that BP is composed of a single phosphorus element and can be degraded into non-toxic phosphate [13,14]. Phosphorus is one of the elements with relatively high content in the human body, accounting for 1 % of the total body weight, second only to calcium, ranking sixth [15]. 85 % of phosphorus exists in the form of hydroxyapatite in bones and teeth, which is necessary for maintaining bone mechanical strength and promoting bone regeneration [16]. Although graphene family materials and MXenes are also emerging materials for bone regeneration, they are not the major constituents of inorganic bone substances and are nondegradable.

The application of BP in enhanced bone regeneration engineering has been widely reported and summarized in recent reviews [[17], [18], [19], [20]]. However, the underlying mechanisms of BP-enhanced bone regeneration have not been fully described. Thus, this review discusses the possible mechanisms of BP-enhanced bone regeneration and the underlying factors involved. This review also provides strategies for further material design of BP-based materials for bone regeneration.

## 2 Physicochemical properties of BP

BP, like graphene, is a two-dimensional (2D) crystal material with a unique layered structure, and the different layers are interconnected by van der Waals forces [5]. From a structural perspective, in a single-layer BP, phosphorus atoms are bonded to neighboring atoms through covalent bonds in sp3 hybridized orbitals, while the interaction between layers is maintained by weak van der Waals forces [12]. Therefore, BP layers can be easily exfoliated into layered 2D black phosphorus nanosheets (BPNSs) from bulk crystals. 0D BP quantum dots (BPQDs) can also be prepared. The main difference between BPNSs and BPQDs lies in the fact that BPQDs belongs to 0D nanomaterials, with higher band gap and surface volume ratio, ultra-small size, and more active edge sites per unit mass [12].

Due to the presence of lone electron pairs in BP, especially BPNSs with fewer layers, is reactive to air and easily degradable. For BP to be widely applied, it is necessary to improve their stability, as the structure and function of BP will significantly decrease or even disappear after oxidation degradation. At present, the main methods for improving the stability of BP are surface protective layer coating, surface chemical modification, and doping [18].

BP exhibit good biocompatibility and degradability, as the physiological byproduct phosphate ions produced during BP degradation is not only harmless, but also a material that participates in bone formation [21]. This endows BP with excellent biocompatibility compared to other nanomaterials (NMs). It has been reported that the cytotoxicity of BP is related to its concentration and size, so the cytotoxicity of BP can be controlled by adjusting the concentration and size used, greatly promoting the application of BP in bone regeneration [22].

BP also exhibit photothermal property and electrical conductivity. As a metal-free layered semiconductor, the BP exhibit a thickness-dependent tunable bandgap ranging from ∼0.3 eV in the bulk to 2.0 eV in the monolayer, which allows BP to have an absorption range spanning both the UV and visible regions, giving them NIR photothermal properties [23]. Due to the tunable gap, BP it can be converted in the two states of insulation and conduction, and the electron migration speed of BP is high.

## 3 Recent advances in applications of BP in bone regeneration

Current applications of BP in bone regeneration are to incorporate BP into substrate materials to obtain an on-demand artificial bone substitutes since the performance of BP alone cannot completely meet the requirements for providing mechanical support of the defect bone and promoting robust osteoblast attachment and differentiation. BP is also an excellent candidate for design of artificial bone substitutes since incorporation of BP promotes bone regeneration, including osteogenesis, angiogenesis and neurogenesis [18].

**Fig. 1:**
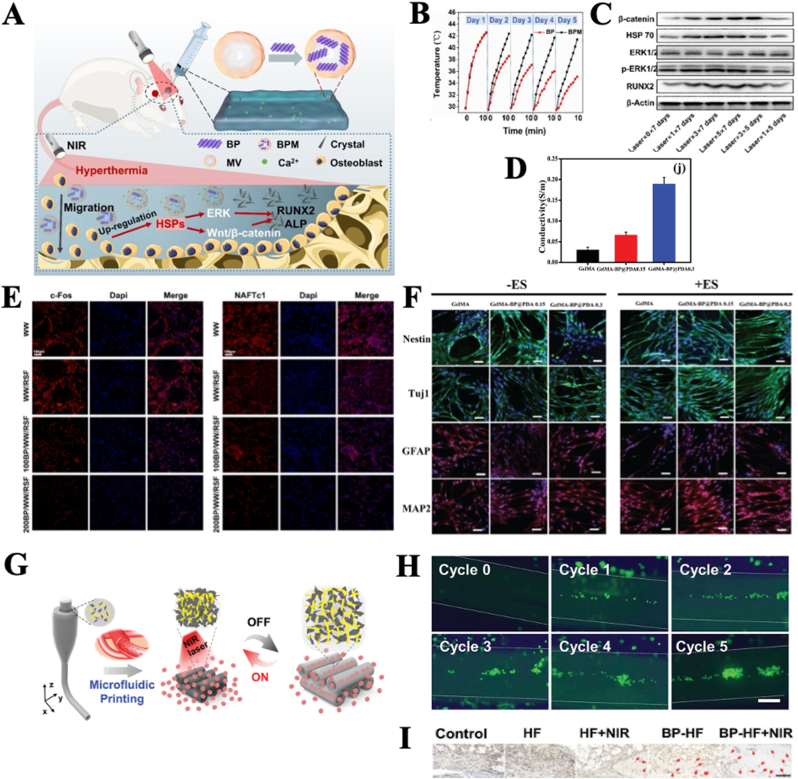
Recent advances in applications of BP in bone regeneration. Fig. 1 A) Schematic illustration of BP-incorporated hydrogel that induces a photothermal effect and mineralization process [7] Copyright 2021 Elsevier. B) Photothermal capacities of BP and MSC-membrane-coated BP (BPM) under NIR irradiation [7] Copyright 2021 Elsevier. C) The relative proteins expression of HSP70, β-catenin, ERK, pERK and RUNX2 of osteoblasts being cultured on BPM-based hydrogels' surface with different time of NIR laser irradiation after culturing for 5 and 7 d [7] Copyright 2021 Elsevier. D) The electrical conductivity of the BP incorporated hydrogel with 0.15, and 0.3 mg mL^−1^ concentrations of BP@PDA [30] Copyright 2020 Wiley. E) Immunofluorescence staining of BP-based scaffold on osteoclast differentiation of monocytes induced by RANKL [29] Copyright 2022 Elsevier. F) Confocal fluorescence micrographs of immunostained cells on BP incorporated hydrogel [30] Copyright 2021 Wiley. G) Schematic illustration of BP-based scaffolds with channels that exhibit shrinkage and swelling behavior controlled by NIR light for bone regeneration [27] Copyright 2021 Wiley. H) The cell proliferation and morphologies of VECs enriched in BP-based scaffold channels [27] Copyright 2021 Wiley. I) Immunohistochemical staining of CD31 [27] Copyright 2021 Wiley.

BP shows great potential to promote osteogenesis due to their unique physiochemical properties. The incorporation of BP into substrates promotes osteogenesis since the degradation product (phosphate ions) of BP can provide raw materials for biomineralization and activate intracellular signal pathways for osteogenesis (typically showed in Fig. 1A) [7,[24], [25], [26], [27], [28]]. In addition, due to the photothermal effect of BP, impart of BP into the substrate induces osteogenesis [7] (typically showed in Fig. 1B–D). The regular and stable local thermal environment created by BP can activate various bone-forming proteins, such as Heat shock proteins (HSPs) and ERK-Wnt/β-catenin-RUNX2 axes, thereby further promoting osteogenesis [7]. Moreover, recent studies showed that BP incorporation obviously increase the electrical conductivity of hydrogels that promote osteogenic differentiation [8]. The incorporation of BP into substrates also promotes osteogenesis by inhibiting osteoclast function probably due to their degradation product [29] (typically showed in Fig. 1E).

BP also shows capacity to promote angiogenesis and neurogenesis, which is mainly attributed to their electrical conductivity [[30], [31], [32], [33], [34]] (typically shown in Fig. 1D–F). Moreover, it was recently discovered the pro-neurogenic properties of BP could be attributed to their effects on regulating redox homeostasis [34], and the pro-angiogenic properties of BP resulted from their regulating of energy metabolism [35]. The pro-angiogenic and pro-neurogenic property of BP were sometimes compensated with other NMs, such as silicon [36] and magnesium [32]. Moreover, utilizing the unique physicochemical properties of BP and optimizing the design of scaffolds can also be options to enhance the pro-angiogenic and pro-neurogenic properties of BP. For instance, a recent study imparts BP into scaffolds with hollow channels, endowing the channels with reversible shrinkage and swelling behavior controlled by NIR light, promoting the penetration of VECs into the scaffold channels (typically showed in Fig. 1G–I) [27].

Ultrasound has been extensively utilized in biomedical fields due to its non-ionizing nature and deep tissue penetration ability [37]. A recent study primarily used ultrasound stimulation to accelerate bone regeneration by causing BP to produce oxygen free radicals to act as an antimicrobial agent, thus benefiting bone regeneration [38]. Also, recent studies are beginning to focus on the immunomodulatory role of BP in bone regeneration. For instance, BP demonstrated their potential to promote bone regeneration through immune modulation [39].

Recent advances in applications of BP in bone regeneration are demonstrated in Fig. 1.

## 4 BP regulates its effects on bone regeneration by influencing the behaviors of bone regeneration-associated cells

Bone regeneration requires the synergistic action of multiple cell types, including MSCs, osteoblasts, osteoclasts, VECs, chondrocytes, and macrophages. Current studies have focused on the role of BPs in bone regeneration by exploring the effects of BPs on these cells. For example, BP is able to induce the osteogenic differentiation of MSCs [8,10], as well as neural differentiation [30,31]. Moreover, BP can promote macrophage polarization from the M1 phenotype toward the M2 phenotype, thereby promoting bone regeneration [9]. BP can also act on VECs to promote angiogenesis [32,33]. How these physicochemical properties of BP interact with various types of cells to promote bone regeneration is the next part of this paper and represents a gap in the current knowledge. The effects of BP on cells involved in bone regeneration are summarized in Table 1, Table 2, Table 3, Table 4.

**Table 1:** Effects of BP on MSCs.

Materials	Design	Effects	Stimulation	Therapy mode	Reference
BPNSs	PLGA/BP scaffold	Promote osteogenesis; Promote osteogenic differentiation of MSCs and upregulation of PI3K-AKT signaling pathway	N/N	Rat model of femoral defects, in vitro BMSC mode	[10]
BPNSs	PVA/CS-MgO-BP hydrogel	Promote osteogenesis; Promote osteogenic migration and differentiation of MSCs and upregulation of PI3K-AKT signaling pathway	NIR	Rat model of cranial defects, in vitro BMSC mode	[48]
BPNSs	BP/HA scaffold	Promote osteogenesis; Promote osteogenic migration and differentiation of MSCs and upregulation of HSP47 and 70	NIR	Rat model of cranial defects, in vitro BMSC mode	[48]
BPNSs	BP/PDA hydrogel	Enhance neurogenesis; enhance neutral differentiation of MSCs and activation of Nestin, Tuj1, GFAP, and MAP2	Electric stimulation	Rat model of subcutaneous implantation, in vitro BMSC mode	[30]
BPNSs	BP/PLCL/Lam nanofiber	Enhance neurogenesis; enhance neutral differentiation of MSCs and upregulation of MAP2	N/N	In vitro BMSC mode	[31]

**Table 2:** The effects of BP on osteoblasts.

Materials	Design	Effects	Stimulation	Therapy mode	Reference
BPNSs	3D BP/MSC membrane hydrogel	Enhance osteogenesis, activate ERK-Wnt/β-catenin-RUNX2 axis	NIR	Rat model of cranial defects, in vitro osteoblast mode	[7]
BPNSs	Injectable BP/CNT hydrogel	Promote osteogenesis; enhance adhesion, proliferation, and differentiation of preosteoblast, and upregulation of BMP-3B and Smad-1	Electric stimulation	Rabbit model of cranial defects, in vitro preosteoblast mode	[8]
BPNSs	3D BP-BG scaffold	Enhance osteogenesis and osteoblastic differentiation of MSCs, release of Pi and secretion of Col-1	NIR	Rat model of cranial defects, in vitro BMSC mode	[79]
BPNSs	PAM/AlgMA/BP hydrogel	Promote the differentiation of proosteoblasts, accelerate the mineralization of the ECM	N/N	Rabbit model of cranial defects, in vitro preosteoblast mode	[47]

**Table 3:** Effects of BP on VECs.

Materials	Design	Effects	Stimulation	Therapy mode	Reference
BPNSs	3D BP/Mg/TCP hydrogel	Enhance early vascularization; enhance cell migration of HUVECs	N/N	Rat model of cranial defects, in vitro HUVEC mode	[32]
BPNSs	BP-containing hollow fibers	Promote osteogenesis and vascularization; enhance migration of HUVECs	NIR	Rat model of cranial defects, in vitro HUVEC mode	[27]
BPNSs	VEGF/BP DNA gel-scaffold	Promote angiogenesis, enhance angiogenic markers expression of HUVECs	N/N	Rat model of cranial defects, in vitro HUVEC mode	[40]
BPNSs	GelMA/BP hydrogel	Promote angiogenesis, activate mitochondrial function and energy metabolism via JAK-STAT-OAS signaling in HUVECs	N/N	Rats skin defects, in vitro HUVEC mode	[93]

**Table 4:** Effects of BP on macrophages.

Materials	Design	Effects	Stimulation	Therapy mode	Reference
BPNSs	Polycaprolactone/BP scaffold	Stimulate macrophages to express iL-33, amplifying the inflammatory response at an early stage, and later promoting the regression of inflammation, promote osteogenic differentiation of MSCs by enhancing IL-33 expression	N/N	Rat model of femoral condyle, in vitro bone marrow macrophages and BMSC mode	[39]
BPNSs	BP suspension	Induce macrophages to release inflammatory factors by activating the NF-κB signaling, and inhibit HO-1 expression	N/N	Mouse intravenous injection, in vitro bone marrow macrophage mode	[118]
BPNSs	PLAG/BP scaffold	Suppress the inflammatory cytokines and promote macrophage polarization to the M2 type, which promotes the proliferation and differentiation of BMSCs via PI3K-AKT signaling	N/N	Rat model of cranial defects, in vitro RAW264.7 cell and BSMC mode	[10]
BPNSs	BP	Activate polarization of M0 macrophages to the M1 phenotype by activating P38 and NF-κB though activation of interaction molecule 2 (STIM2) and facilitate Ca2^+^ influx	N/N	In vitro RAW264.7 cell mode	[114]

### 4.1 Mesenchymal stem cells (MSCs)

#### 4.1.1 Enhanced osteogenic differentiation of MSCs

The osteogenic differentiation of MSCs is one of the fundamental processes of osteogenesis. Studies have shown that BP can induce the osteogenic differentiation of MSCs [40,41]. We assume the mechanism by which BP induces osteogenic differentiation of MSCs via regulating cell cytoskeleton remodeling.

BP can regulate cytoskeletal remodeling in MSCs to promote their osteogenic differentiation. Extracellular mechanical signals (e.g., stiffness, heat and electrical signals) can be transmitted to the cytoskeleton through mechanotransduction, causing changes in the activity of relevant ion channel receptors, which are then transmitted to the nucleus, where they activate mechanotransduction signaling pathways, for example BMP/SMAD and PI3K/AKT signaling. It has been demonstrated that within the stiffness threshold, a stiffer substrate facilitates MSC stretching that promotes osteogenic differentiation and inhibits lipogenic differentiation of MSCs [[42], [43], [44], [45]]. BP has a high stiffness (modulus of elasticity: C11 = 189 GPa, C22 = 58 GPa, C33 = 52 GPa, breaking strength: 2.1 GPa) [46] and can form chemical bond with hydrogels, as well as generate friction between the BP and hydrogel matrix for its 2D shape and rough surface. Thus, BP were reported be an enhancer to increase the stiffness of the substrate [47]. Furthermore, BP could promote and osteogenic differentiation of MSCs via activation of PI3K/AKT signaling [48]. Therefore, we suggest that BP may increase the stiffness of the substrate to modulate cytoskeleton remodeling in MSCs and thus their osteogenic differentiation. HSP activation leads to degradation of the basement membrane by upregulating matrix-degrading metalloproteinase (MMP), such as MMP-2 and MMP-9, as well as the downstream osteogenic signalings [49,50]. Under NIR light, BP promoted MSC migration and differentiation via activation of the HSP (i.e., HSP47 and HSP70)-mediated MMP and ERK-Wnt/β-catenin-RUNX2 axes [7,48,51,52]. Therefore, we speculate that the photothermal properties of BP could promote the osteogenic differentiation of MSCs by regulating cytoskeletal remodeling. Studies showed the electroactive materials could induce osteogenic differentiation of MSCs mediated by opening of Ca^2+^, which further stimulate actin remodeling together with downstream mechano-signaling like BMP/SMAD signaling [[53], [54], [55]]. BP exhibits a high electrical conductivity (up to 300 S cm^−1^) [56]. Moreover, BP were reported to increase the osteogenic differentiation of MSCs via activating BMP2/SMAD5 signaling pathway [57]. Therefore, we speculate that the electrical conductivity of BP could promote the osteogenic differentiation of MSCs by regulating cytoskeletal remodeling and downstream mechanotransduction signaling pathway activation. However, the above conjecture remains to be confirmed by further studies.

In additional, BP may promote the osteogenic differentiation of MSCs by adsorbing and releasing pro-osteogenic molecules. GO enhances BMP2 expression and adsorbs endogenous bone morphogenetic protein 2 (BMP2), which enhances the retention of endogenous BMP2 and the subsequent release of BMP2 to promote the osteogenic differentiation of MSCs. BP and graphene are two-dimensional materials, and BP has a high affinity for biomolecules, which makes it an excellent molecular carrier [58]. For instance, it was shown that BP binds to BMP2 via electrostatic adsorption and is capable of achieving controlled long-term release of BMP2 [26]. Furthermore, studies have also shown that BP can upregulate the expression of BMP2 [10,41]. Therefore, can BP promote the osteogenic differentiation of MSCs by adsorbing and releasing endogenous pro-osteogenic molecules, such as BMP2 and TGF-β? This will also be an idea for future research [59].

#### 4.1.2 Enhanced neural differentiation of MSCs

Nerve network construction during bone healing plays an essential role since the nerve in the periosteum activates and regulates the process of osteogenesis. Studies have shown that BP can induce neurodifferentiation in MSCs, characteristics of elevated expression of growth associated protein 43 (GAP43) and microtubule associated protein 2 (MAP2) [[32], [33], [34]]. We suggest that the electrical conductivity, antioxidant properties and surface morphology of BP may induce neural differentiation of MSCs by regulating cytoskeletal remodeling.

The electrical conductivity of BP may promote neural differentiation of MSCs by regulating Ca^2+^ channels and actin remodeling. Electrically conductive materials can mimic the natural bioelectricity that open Ca^2+^ channel [53,60,61], which further stimulate actin remodeling together with downstream signaling, like PI3K-AKT signaling, as well as neurotrophin secretions (such as NGF and BDNF), that enhance the neural differentiation of MCSs [62,63]. BP exhibits high electrical conductivity [56] and its incorporation significantly increased the electrical conductivity of substrates that open calcium ion channels and enhanced the neural differentiation of MSCs via activating AKT signaling [64], as well as the promote the expression of NGF and BDNF [30,31,65]. The extinguishing property of BP is its anisotropic electrical conductivity, but it has always been ignored in the application of BP for designing materials for bone treatment. Aligned scaffolds with anisotropic electrical conductivity were superior to scaffolds with conductivity in nerve regeneration in all directions [66]. To make full use of the anisotropic electrical conductivity of BP, the alignment of BP into the substrate can be controlled with an external magnetic field.

BP may also promote the neural differentiation of MSCs via regulating redox homeostasis. Recent findings suggest that BP can promote the neural differentiation of neural progenitor cells (NPCs) by activating the Nrf2 signaling pathway and redox homeostasis [34]. Studies have shown that Nrf2 regulates redox homeostasis and regulates energy metabolism in ganglionic neurons and astrocytes [67]. The ability of BP to regulate redox homeostasis should be related to the ability of BP to savage ROS and the ROS savaging ability of BP have been reported in a few studies [68,69]. Whether BP can regulate energy metabolism of MSCs by mediating the Nrf2 signaling pathway requires further investigation. However, other studies reported that low concentrations of BP (7.10 μg/mL) could induce ROS generation and obvious cell death in several cell lines [70,71]. The different effects of BP on cells might be attributed to differences in BP thickness. Generally, few-layered BP acts as a ROS scavenger, while multilayered BP acts as a ROS inducer.

The surface morphology of BP may also promote the neural differentiation of MSCs. Stem cells have been reported to successfully differentiate into mature neurons or glial cells when grown on anisotropically patterned substrates [72,73]. The surface topography regulates the neural differentiation of stem cells by modulating cytoskeletal hyperremodeling [74,75]. BP has an anisotropic surface topography. However, the surface topography of BP may not play a very important role in promoting the neural differentiation of MSCs since stiffness plays a dominant role in the neural differentiation of MSCs. Soft substrates are preferred for the neural differentiation of stem cells [74,76].

To summarize, given that insufficient innervation of bone repair materials remains a challenge, the design of BP-based materials should make full use of their pro-neurogenic properties. The anisotropic electrical conductivity of BP should be fully utilized. In addition, research should also focus on the possibility that BP promotes neural differentiation by regulating redox reactions.

**Fig. 2:**
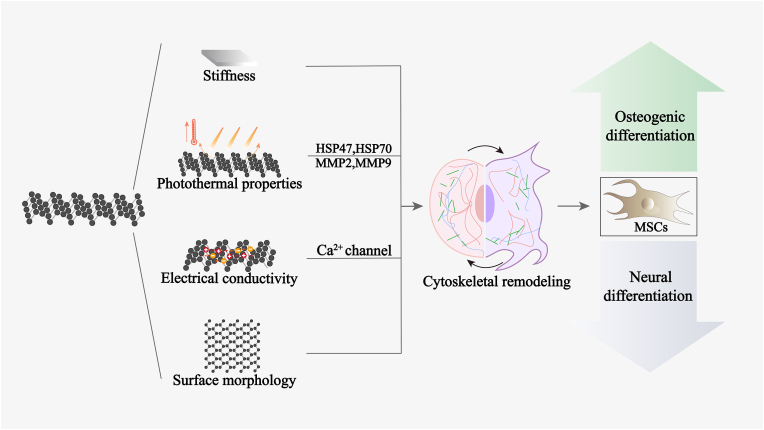
Illustration of mechanisms of BP-promoted differentiation of MSCs.

The mechanisms of BP-induced differentiation of MSCs are summarized in Fig. 2.

### 4.2 Osteoblasts

After bone defects, osteoblasts migrate to the defect area and synthesize ECM and hydroxyapatite, and hydroxyapatite is deposited into the organic matrix to form mineralized new bone tissue. Studies have confirmed that BP promotes osteogenic differentiation and biomineralization of osteoblasts [7,77]. BP may promote the differentiation of osteoblasts by regulating cytoskeletal remodeling. BP also provides Pi source for biomineralization.

The photothermal properties and electrical conductivity of BP can contribute to osteoblast differentiation by regulating cytoskeletal remodeling. BP can induce mild photothermal effects under NIR light, which activates HSP-mediated MMPs and the ERK-Wnt/β-catenin-RUNX2 axis [78]. Its activation and subsequent degradation are followed by cytoskeletal remodeling, ultimately leading to osteoblast migration and differentiation. In addition, the electrical conductivity of BP promoted the differentiation of osteoblasts. In response to electrical stimulation, the conductive material prompts actin remodeling and the activation of downstream mechanotransduction signaling pathways, such as the integrin-mediated BMP/SMAD signaling pathway [[53], [54], [55]]. Due to the high electrical conductivity of BP [124], the addition of BP to a substrate under electrical stimulation significantly increases its conductivity and promotes the differentiation of osteoblasts and the expression of BMP3B and SMAD1 [8].

BP is also able to promote osteoblast mineralization. We believe that the process by which BP promotes osteoblast mineralization is as follows. Primarily, BP enhances the secretion of Col-1 by osteoblasts by activating intracellular signals, such as ERK and PI3K signaling [39,79]. Next, in osteoblasts, an increased abundance of PO_4_^3+^ degraded from BP promotes the formation of PO_4_^3+^ clusters on ER membrane. The PO_4_^3+^ and Ca^2+^ clusters are transported from the ER to mitochondria to form amorphous calcium phosphate precursors [80]. The biomineral precursors were thereafter transported to the extracellular space for intrafibrillar mineralization.

BP easily degrades when exposed to oxygen and water and even degrades before implantation due to the presence of lone pairs of electrons in the phosphorus atom of BP [81]. Thus, BP is expected to also be degraded extracellularly before being captured by cells. To form phosphate clusters, extracellularly degraded BP requires extra energy, and the Na/Pi cotransporter must be transported into cells [82]. Therefore, we assume that stable BP that achieves intracellular degradation is preferable for biomineralization. At present, the main methods for improving the stability of BP are surface protective layer coating, surface chemical modification, and doping [18]. The degradation products of BP also include PO_2_^3−^ and PO_3_^3−^ [83]. However, their role in biomineralization remains unclear. It was reported that the interchange of PO_2_^3−^, PO_3_^3−^ and PO_4_^3−^ in oxygenated water is prohibited [84]. In the circulation of PO_2_^3−^ and PO_3_^3−^, there may be chances for PO_2_^3−^ and PO_3_^3−^ to transit to PO_4_^3−^ by intestinal bacteria since it was shown that PO_2_^3−^ and PO_3_^3−^ can be oxidized into phosphate by microbes to obtain a Pi source [85]. These transitions are expected to require additional biological processes and energy. Thus, increased degradation products of PO_4_^3−^ by BP should be achieved for the benefit of biomineralization.

In summary, the photothermal effect, electrical conductivity, and metabolites of BP may promote osteoblast differentiation and mineralization.

**Fig. 3:**
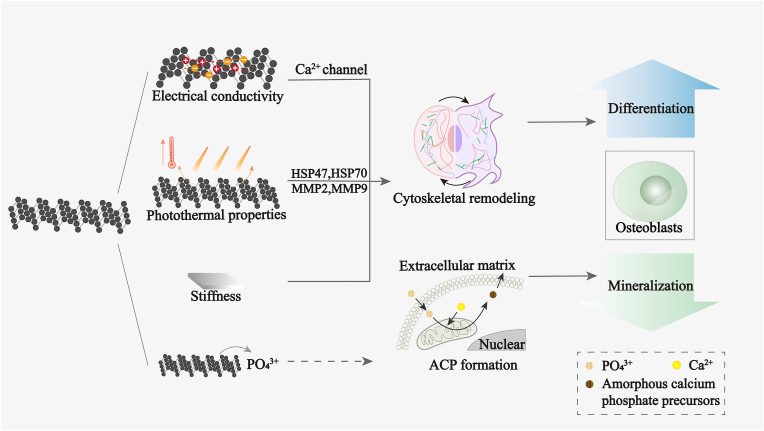
Illustration of mechanisms of BP-promoted differentiation of osteoblasts.

The mechanisms of BP-induced differentiation of osteoblasts are summarized in Fig. 3.

### 4.3 Osteoclasts

Bone resorption by osteoclasts plays an important role in skeletal homeostasis. During this process, osteoclasts undergo continuous cytoskeletal remodeling. Reportedly, BP can inhibit osteoclast differentiation by depressing the NF-κB ligand (RANKL)–RANK receptor interaction [86]. However, the exact underlying mechanism of BP-induced osteoclast differentiation is still unclear. On the one hand, the metabolites of BP may inhibit osteoclast differentiation via NPT2A-dependent inhibition of RANK–RANKL signaling [87]. On the other hand, the strong attraction to the membrane-cytoskeleton structure of BP may also inhibit osteoclast differentiation. It was reported that fullerene can attach to the surface of F-actin, disrupt actin dynamics and inhibit RANKL–RANK signaling [86]. Similarly, BP is also highly adsorptive to cell membrane skeletal structures [88]. The addition of BP was reported to cause cytoskeleton-related protein expression changes [86]. Thus, we assumed that BP may attach to the membrane-cytoskeleton structure that distributes the membrane-cytoskeleton structure, thus inhibiting osteoclast differentiation. The assembly and disassembly of osteoclast pseudopod vesicles, which can drive cytoskeletal rearrangements, play an important role in regulating osteoclast activity. Whether pseudopod vesicles play a role in BP-mediated osteoclast inhibition requires further investigation.

### 4.4 Chondrocytes

Engineering bone through the endochondral pathway has gained increased attention in recent years. During endochondral ossification, chondrocytes are formed first, and then, the chondrocytes are gradually replaced by osteocytes [89]. BP exerts a protective effect on chondrocytes mainly by scavenging free radicals. Reportedly, BP restored the mitochondrial function of chondrocytes via scavenging ROS [68]. Moreover, BP is very likely to enhance osteogenic differentiation of MSCs via modulating the energy metabolism of chondrocytes. This might be resulted from the stiffness of BP since it was reported the increased substrate stiffness could contribute to enhanced oxidative phosphorylation (OXPHOS) of mitochondria of chondrocytes that facilitate tissue regeneration [90].

### 4.5 Vascular endothelial cells (VECs)

Angiogenesis is an essential part of bone regeneration. BP was proven to promote angiogenesis [33]. However, the mechanism by which BP induces angiogenesis remains unclear. We believe that BP promotes angiogenesis by degrading the basement membrane and promoting the proliferation of VECs, as well as regulating the metabolism of VECs.

VECs can be activated by angiogenic factors (e.g., VEGF, FGF, or chemokines). Activated VECs are called tip cells, and they release enzymes that degrade the basement membrane, allowing VECs to migrate out of existing vessels and thus begin to sprout. As the VECs of the tip cells proliferate, they stimulate the continued extension of the vessel and the formation of new vessels [91,92]. BP promotes angiogenesis and the upregulation of angiogenic factors, i.e., VEGF, FGF, CXCL8, CCL20, and IL-17A [40,93], as well as MMPs in VECs [93]. Furthermore, BP promoted the migration and proliferation of VECs and the activation of ERK and PI3K/AKT signaling [94,95]. Thus, we hypothesized that BP promotes angiogenesis as follows: BP promotes the secretion of angiogenic factors (VEGF, FGF, CXCL8, CCL20, and IL-17A) from VECs that lead to MMP-mediated degradation of the basement membrane, causing VECs to migrate out of existing vessels and thus begin to spout [94]. Next, BP activated ERK, and PI3K/AKT signaling promoted the proliferation of VECs in tip cells, continued extension of the canister wall, and subsequent formation of new blood vessels. However, the entire route by which BP promotes angiogenesis is not clear, and there are many gaps. For example, it is not clear how BP induces the secretion of VEGF and FGF. BP was reported to activate HIF-1α secretion in VECs [93], and HIF-1α was reported to further induce the secretion of VEGF [96]. HIF-1α may be triggered by mild amounts of ROS produced by BP.

In addition, BP can promote angiogenesis by regulating the metabolism of VECs to enhance cellular function. Angiogenesis is dependent on the energy metabolism of VECs. A very recent study reported that BPs promote angiogenesis, which is likely attributed to the enhanced OXPHOS of VECs [93]. BP-induced OXPHOS was attributed to metabolites of BP and BP-generated mild ROS since low doses of NMs have been reported to induce moderate ROS increases and lead to adaptive responses in biological systems [97]. However, VECs rely on glycolysis to produce >85 % of their energy, as well as adenosine triphosphate (ATP), which is far greater than that of most other quiescent cells [98]. In VECs, tip cell-induced VEGF stimulates glycolysis by upregulating the expression of PFKFB3 and HK2 [99]. As discussed previously, BP induces VEGF production, so could BP promote angiogenesis by regulating glycolysis via regulating PFKFB3 and HK2in VECs? This needs to be further investigated.

BP enhanced angiogenesis could further facilitate osteogenesis. It was recently showed VECs are reduced in tissues with critical-size bone defects. Increased angiogenesis at the site of bone defect will stimulate osteogenesis [100]. Angiogenesis can promote osteogenesis not only through the supply of oxygen and nutrients but also through the interactions between VECs and bone cells. For instance, VEGF and FGF enhance osteogenic differentiation by activating the PI3K/AKT and β-catenin pathways in MSCs and osteoblasts [[101], [102], [103], [104], [105]], as well as by providing a local microenvironment via paracrine signal release or immune modulation [104,106,107].

In conclusion, research on the role and mechanism of BP in angiogenesis is still at a preliminary stage. Future studies could focus on the role and mechanism of BP-induced enhancement of angiogenesis by energy metabolism in VECs.

**Fig. 4:**
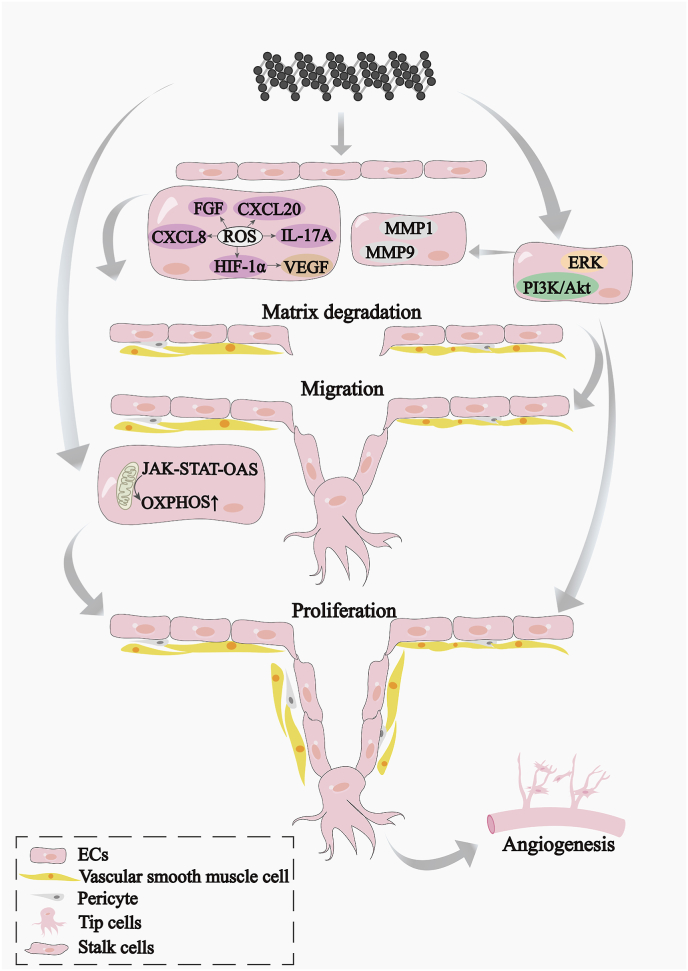
Illustration of mechanisms of BP promoted angiogenesis by VECs.

The mechanisms by which BP promotes angiogenesis via VECs are summarized in Fig. 4.

### 4.6 Macrophages

In the early stage of bone defects, there is a continuous inflammatory state, which is mainly caused by macrophages remaining in the M1 phenotype and continuing to secrete proinflammatory factors, and the continuation of inflammation is the reason for hindering bone regeneration [10,108]. M2 macrophages can secrete anti-inflammatory inhibitory factors to promote bone regeneration in the bone defect area [109,110]. Therefore, efficient and timely promotion of macrophage polarization from the M1 phenotype to the M2 phenotype can promote bone regeneration. BP can regulate bone regeneration by targeting the immunomodulation of macrophages, providing a new strategy for the treatment of bone regeneration. Reportedly, BP stimulates macrophages to amplify the inflammatory response at an early stage and promotes the regression of inflammation, resulting in a beneficial immune environment that facilitates bone regeneration [39].

#### 4.6.1 Promotion of M1 macrophage polarization

During the initial inflammatory phase of bone regeneration (within 3–4 d after defect induction), macrophages in the defect area are mostly of the M1 phenotype (approximately 85 %) and secrete cytokines (i.e., L-1β, IL-6, VEGF and TNF-α) for host defense, amplify inflammatory reactions and recruit MSCs and osteoblasts [10,111,112]. This transient inflammation is thought to favor bone repair. During the initial stage of bone regeneration (within 3 d after bone defect), BP induces an innate immune response and M1 polarization of macrophages [113]. BP-induced ROS generation and Ca^2+^ influx that activate P38 MAPK and NF-κB signaling, leading to macrophage polarization from the M0 to the M1 phenotype and subsequent M1 macrophage cytokine release (i.e., IL-1β, IL-6, IL-8, IL-12, TNF-α and INF-γ) [[114], [115], [116], [117], [118]]. Moreover, BP-generated ROS can also inhibit anti-inflammatory molecules such as heme oxygenase-1 (HO-1), leading to the aggravation of inflammation [118]. Moreover, BP can induce IL-33 expression [39], possibly resulting from BP-generated ROS, which bind to the ST2 receptor, thereby activating the downstream NF-κB and MAPK pathways.

From this, we can infer that the application of BP at the early stage of bone defects can promote the development of the inflammatory phase of bone defect healing, but few studies have focused on this, probably because the inflammatory phase is very short, and poorly controlled doses of BP may lead to prolonged inflammation. To address this issue, we can use BP as a proinflammatory material. To avoid prolonged inflammation, the dose of BP should be controlled to achieve a short-term effect, and the actual effect needs to be confirmed by further experiments.

#### 4.6.2 Promotion of M2 macrophage polarization

It has been reported that in the reconstruction n stage of bone regeneration, the application of BP can induce macrophage polarization from the M1 to M2 phenotype, as evidenced by the elevation of M2 macrophage markers (Arg-1 and CD206) and M2 macrophage factors (IL-10, IL-4, and TGF-β) and a decrease in the expression of M1 macrophage markers (iNOS and CD86) and M1 macrophage cytokines (IL-1β and TNF-α) [9,10]. However, the mechanism by which BP induces M2 macrophage polarization remains unclear. We suggest that the possible mechanisms of BP-induced M2 macrophage polarization include the modulation of the macrophage skeleton and the enhancement of mitochondrial energy metabolism in macrophages.

The stiffness of the material is critical for modulating the M1 and M2 phenotypes of macrophages since material stiffness modulates the remodeling of the macrophage skeleton through mechanotransduction [44,119]. Studies have shown that stiffer substrate materials promote macrophage stretching (macrophages are rounded on less stiff bases, whereas they are spread out and square on stiffer substrates). Elevated macrophage stretching on the substrate surface facilitates macrophage polarization from the M1 phenotype to the M2 phenotype [120,121]. The addition of a small amount of BP to the hydrogel significantly enhanced the stiffness of the hydrogel (from 0.5 to 2 MPa) and promoted macrophage polarization from the M1 to M2 phenotype in the bone defect area [9]. Therefore, we speculate that the high stiffness of BP may promote macrophage polarization from the M1 to M2 phenotype by modulating macrophage skeleton remodeling and facilitating macrophage extension on the basal surface. In addition, other physicochemical properties of BP, such as its high hydrophilicity and rough surface, may also promote macrophage stretching, which may induce macrophage polarization from the M1 to M2 phenotype.

BP may trigger M2 macrophage polarization by enhancing mitochondrial energy metabolism. It was recently reported that IL-33 can be recognized by macrophages to initiate UCP2-mediated mitochondrial metabolic reprogramming (mitochondrial uncoupling), resulting in increased levels of the metabolite itaconic acid and upregulation of the expression of the transcription factor GATA3, thereby promoting macrophage polarization from the M1 to the M2 phenotype [122]. It has been reported that BP can promote osteogenic differentiation by activating IL-33 in macrophages at later stages of inflammation resolution [39]. But how does BP cause IL-33 release? Studies have reported that Caspase-11 induces the shearing of intracellular Gasdermin D to form holes in the cell membrane, thereby facilitating the release of IL-33 from the cytoplasm to the outside of the cell [123]. Caspase-11 can be activated by activated inflammasomes [124,125]. Therefore, we hypothesize that BP may act as an exogenous agent to activate the inflammasome directly or by generating a small amount of ROS, thereby prompting Caspase-11 to induce intracellular Gasdermin D to undergo shearing to form holes in the cell membrane, thus facilitating the release of cytoplasmic IL-33 into the extracellular compartment. This conjecture remains to be verified by future studies.

To summarize, current studies reveal the immunomodulatory potential of BP in bone regeneration through the regulation of both early-stage inflammatory responses and later-stage inflammation resolution. Premature induction of macrophage polarization toward the M2 phenotype during the inflammatory phase may be detrimental to bone regeneration. However, BP-induced macrophage polarization toward the M2 phenotype, which begins 7 d after bone defects [10], does not interfere with the role of M1 macrophages during the inflammatory phase.

**Fig. 5:**
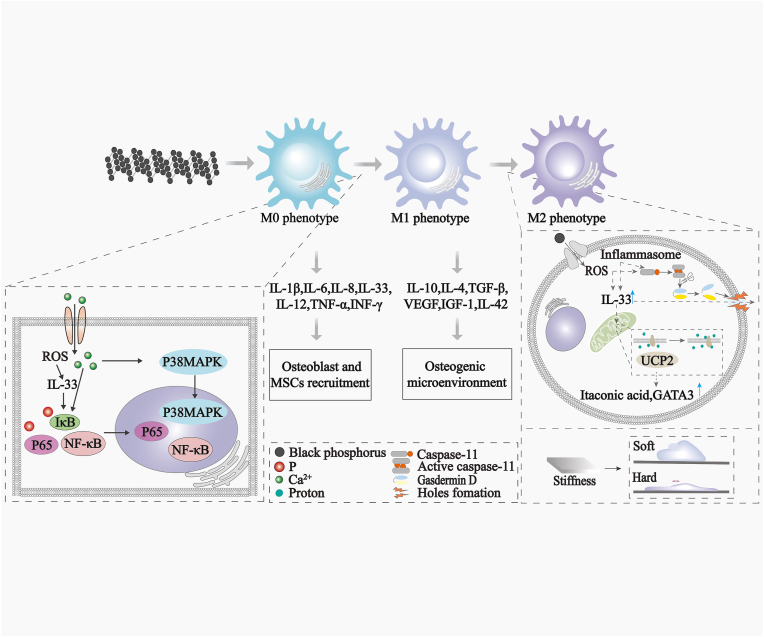
Illustration of mechanisms of BP-induced macrophage polarization.

The mechanisms of BP-induced macrophage polarization are summarized in Fig. 5.

Other immune cells, such as neutrophils, NK cells, and T cells, also play important roles in bone regeneration. During the acute phase after bone defects, activation of neutrophils triggers inflammation, and activation of pro-inflammatory T cell subtypes (such as Th1, Th17, and CD8^+^ T cells) can further amplify the inflammatory response [[126], [127], [128]]. Meanwhile, NK cells can rapidly activate pro-inflammatory T cells, promoting their proliferation and cytokine production, thus amplifying the inflammatory response. Activation of Th2 and regulatory T cells (Tregs) can help alleviate acute inflammation. Apart from macrophages, the roles of BP and other immune cells have been less reported. Limited studies have shown that BP can activate NK cells through metabolic reprogramming, thereby amplifying the inflammatory response [129]. Whether BP can interact with other immune cells to alleviate inflammation after the acute phase has not been reported. Future research could focus on the interactions between BP and these immune cells, especially in terms of alleviating inflammation.

## 5 Factors influencing the effects of BP on bone regeneration

The physiochemical properties of BP (including size, shape, surface charge and hydrophilicity) critically influence its effects on bone regeneration.

### 5.1 Degradability of BP

The degradability of BP ensures its ability to promote bone regeneration. BP is degraded both intracellularly and extracellularly into nontoxic phosphate ions, PO_4_^3+^, which together with Ca^2+^ clusters to form amorphous calcium phosphate precursors, which are transported to the extracellular space for intrafibrillar mineralization [80]. The degradation products of BP may also modulate the local microenvironment through the recruitment of endogenous cells, including immune cells and MSCs. Moreover, the degradability of BP ensures its ability to promote bone regeneration by generating dynamic modulation function on that generate a preferred environment for bone regeneration [130]. The sustained release of PO_4_^3+^ for more than 4–6 weeks is required for biomineralization since initial mineralization mainly begins at 4–6 weeks after bone defect induction and is completed over the course of several months [131]. Nevertheless, BP easily degrades upon exposure to oxygen and water, and partial degradation occurs at the initial stage of implantation or even before implantation. A BP with a thickness of 3.6–19.9 nm is completely degraded within 10 d of exposure to oxygen and water [132]. Thus, the rapid degradation of BP nanoparticles significantly affects their structure, electronic properties, and biomedical function. Moreover, the rapid degradation of BP results in an acidic microenvironment that may cause osteoclastogenesis.

The conventional method to retard the degradation of BP is to add a polymer coating to the surface, and the main binding modes to the polymers include electrostatic adsorption and covalent conjugation. The negatively charged surface of BP can be easily bound to cationic polymers, such as amino polyethylene glycol (PEG-NH2), polyethyleneimine (PEI) and polydopamine (PDA), by electrostatic adsorption [133,134]. Electrostatically adsorbed polymer coatings are susceptible to delocalization, whereas covalently conjugated coatings (e.g., using aryl diazonium chemistry to form P-C covalent bonds on the BP surface) rarely delocalize [135]. In addition, natural cell membrane or vesicle membrane coating is another effective option for BP surface modification because it not only ensures the stability of BP but also acts as a strong camouflage that can target specific cells.

Although the degradation mechanism of BP in a typical environment is relatively precise, the biosafety of BP is difficult to ensure in the complex microbial environment of organisms. Therefore, the degradation mechanism of BP in the body needs further research and confirmation.

### 5.2 Shape

The shape of NMs plays a critical role in nano-biological interactions [136,137]. However, no study has compared the effects of BPNSs and BPQDs in promoting bone regeneration. Compared with BPQDs, 2D sheets are more conducive to cell adhesion and extension because of their larger superficial area, which is beneficial for promoting the osteogenic differentiation of MSCs. Moreover, BPNSs might be more favorably taken up than BPQDs because of their higher aspect ratio (AR) [137] and sharp edge [138]. Increased cellular internalization leads to improved bone regeneration. However, BPQDs exhibit better photothermal properties than BPNSs, probably due to their different edge structures [139].

### 5.3 Surface charge

The surface charge of NMs impacts bone regeneration [140]. This is because the surface charge of NMs plays an essential role in nano-cell interactions, such as cellular uptake and protein binding [141,142]. Whether the surface charge of BPs has an impact on bone regeneration has not been fully studied. BP exhibits a negative surface charge because POx is generated from degradation and free lone pair electrons [116,117]. Corona formation in the blood does not change the negative surface charge since more than 62 % of the components of the protein corona on BP in contact with the blood are negatively charged proteins [114,117]. The formation of a protein corona around the BP surface leads to a “normalization” of the zeta potential (from −18.1 to −8.4 Mv) [117].

The negative surface charge of BP is probably more beneficial for bone regeneration than the positive surface charge. This is mainly because positively charged NMs induce more severe toxicity effects than negatively charged and neutral NMs since they perturb the continuity of the negatively charged plasma membrane and are more likely to be taken up by macrophages [130,[143], [144], [145]]. However, it has been reported that the cellular uptake efficacy of BP improves significantly with increasing surface charge, and BP-based NMs with a zeta potential of −7.78 mV almost completely block their cellular uptake [146]. However, considering that BP easily degrades before cell uptake and that the degradation products can facilitate bone regeneration, negative surface charge-induced decreases in cellular uptake might not play an essential role in BP-induced bone regeneration.

To date, the understanding of the effect of the surface charge of BP on bone regeneration remains in the primary stage. For instance, the surface charge of BP is involved in possible electron transfers between BP and biomolecules as well as in possible transfers with biological structures, such as organelles [147]. The surface charge also impacts the intracellular localization of NMs [148]. However, the impact of surface charge-dependent electron transfer of BP and the intracellular localization of BP on bone regeneration remain unknown.

### 5.4 Size

The size of BP plays a critical role in determining its effect on bone regeneration since different sizes induce varied BP-bio interactions. For instance, an in vitro study showed that a smaller BP (18.8 nm) was better at enhancing the migration, proliferation and osteogenic differentiation of preosteoblasts than a larger BP (107.1 and 242.3 nm) [132]. Smaller BP is more likely to promote bone regeneration since it more likely to be taken up by bone regeneration related cells [149], cause less damage to cell structure [150] and induce fewer immune effects [117,151]. However, large BP materials exhibit greater photothermal conversion efficiency than small BP materials [152]. The higher efficiency for photothermal conversion of larger BP should be attributed to their greater surface area. Moreover, NMs >100 nm in size are more readily taken up by macrophages, and the larger the NM are, the stronger the interaction with the macrophages [149].

To summarize, the current understanding of the size effects of BP on bone regeneration is in an early stage. The type of protein adsorbed on the BP surface is directly related to the size of the BP [115]. Therefore, what is the effect of differences in the size of protein crowns on bone regeneration? This needs to be further investigated.

### 5.5 Hydrophilicity

BP was reported to be hydrophilic due to its strong out-of-plane dipolar moment [34]. The hydrophilicity of BP regulates its effects on bone regeneration by influencing the behaviors of bone regeneration-associated cells and moderating immunity. Hydrophilic surfaces exhibit high cell adhesion and proliferation [153], an extended circulation period [154] and the ability to induce M2 macrophage phenotype polarization [155]. In contrast, hydrophobic surfaces exhibit low cell adhesion and proliferation, limited circulation time because of phagocytosis, and induction of a proinflammatory M1 macrophage phenotype [[153], [154], [155]].

To make full use of its hydrophilic nature, the surface modification of BP with hydrophilic polymers has been used to enhance the hydrophilicity of BP. For example, hydrophilic functionalized PEG, poly (diallyldimethylammonium chloride) (PDDA), poly(lactic-co-glycolic acid) (PLGA), and PEI are polymers commonly used to modify BP surfaces due to their advantages such as fair hydrophilicity, biocompatibility and physiological stability in media [133,153,156,157].

## 6 Conclusions and outlooks

BP has shown good potential for promoting bone regeneration. BP regulates the behavior of MSCs, osteoblasts, VECs, macrophages, and other cells to promote bone regeneration mainly through the regulation of cytoskeletal remodeling, energy metabolism and redox homeostasis, etc. In addition, moderating the physicochemical properties of BP (i.e., shape, size, surface charge and hydrophilicity) can alter the effects of BP on bone regeneration. This review reveals the underlying mechanisms of BP-enhanced bone regeneration and provides strategies for further material design of BP-based materials for bone regeneration.

At present, the mechanism by which BP promotes bone regeneration has not been fully clarified and will be the direction of future research. The ability of BP to promote bone regeneration is derived from the properties of BP, such as hardness, surface morphology, electrical conductivity, photothermal effect, oxidation resistance and surface adsorption properties. The effect of antioxidant and surface adsorption properties of BP on bone regeneration has been less studied. More attention should be paid to aspects above in the future. Moreover, it is a challenge to adapt the degradation rate of BP to the bone regeneration rate. The complete degradation of BP more than 4–6 wk favors bone regenerative repair.

Current studies have shown the potential of BPs to regulate cellular energy metabolism, which can regulate tissue regulation. For example, BP is able modulate macrophage polarization in order to exert an immunomodulatory effect, thereby promoting bone regeneration [9,10]. However, research on the role and mechanism by which BP regulates cellular energy metabolism is still at a preliminary stage. Future studies should focus on changes in cellular metabolism by performing as well as metabolomics testing. If changes in metabolism occur, further gene or protein sequencing should be done to find the reason why BP works by regulating cellular metabolism in order to promote bone regeneration.

The toxicity of BP is a factor in its bone regeneration effect. Studies have shown that BP exhibits toxic effects [19,71,158,159]. However, current studies on the toxic effects induced by BP have not been fully elucidated. Particularly, most of the available toxicological studies on BP have adopted limited exposure times, as most are usually less than 7 d, mainly representing acute (up to 14 d) exposure. However, subacute (up to 28 d), subchronic (up to 90 d) and chronic (up to 4 months) toxicology studies of BP remain challenging. Furthermore, little is known about the underlying mechanism of BP-induced toxicity. For instance, what kind of diseases can be caused by the toxic effects of BP? Thus, in-depth studies on the toxic effects and the underlying mechanism of BP are needed in the future.

## Ethics approval and consent to participate

This dissertation is a review and does not deal with human-animal studies.

## Consent for publication

Not applicable.

## Availability of data and materials

All data generated or analyzed during this study are included in this published article.

## Funding

This work was supported by the Guangdong Medical Science and Technology Research Fund Project (A2022358).

## CRediT authorship contribution statement

**Ting Sun:** Writing – review & editing, Writing – original draft. **Chufeng Li:** Visualization. **Jiayi Luan:** Data curation. **Fujian Zhao:** Writing – review & editing, Methodology. **Yanli Zhang:** Validation. **Jia Liu:** Writing – review & editing. **Longquan Shao:** Supervision.

## Declaration of competing interest

The authors declare that they have no known competing financial interests or personal relationships that could have appeared to influence the work reported in this paper.

## Data Availability

No data was used for the research described in the article.

## Abbreviations

MSCs: mesenchymal stem cells
VECs: Vascular endothelial cells
BP: black phosphorus
NMs: nanomaterials
BPNSs: black phosphorus nanosheets
BPQDs: BP quantum dots
HSPs: heat shock proteins
MMP: mediated matrix metalloproteinase
NIR: near-infrared
NPCs: neural progenitor cells
OXPHOS: oxidative phosphorylation
RANKL: NF-κB ligand
ATP: adenosine triphosphate
PEG-NH2: amino polyethylene glycol
PEI: polyethyleneimine
PDA: polydopamine
PEG: polyethylene glycol
PDDA: poly (diallyldimethylammonium chloride)
PLGA: poly (lactic-co-glycolic acid)
GAP43: growth associated protein 43
MAP2: microtubule associated protein 2
NGF: nerve growth factor
BDNF: brain derived neurotrophic factor
BMP2: Bone morphogenetic protein 2
AR: aspect ratio
